# Report on Sanitary Characteristics of Chicago

**Published:** 1855-11

**Authors:** N. S. Davis


ART. II.—*Report on the Sanitary Characteristics of Chicago during the Summer of* 1855 *; or the connection between Meteorological Phenomena and the Prevalence of certain diseases.* By N. S. DAVIS, M. D., &c. Read to the Cook County Medical Society, October 9th, 1855.

THERE is no subject of more interest to the physician, or of greater importance to the community, than that which relates to the causes of disease. More especially is this true in relation to those causes, whether predisposing or exciting, which determine the prevalence and severity of such endemic and epidemic diseases as recur annually, at stated seasons, or more irregularly and at longer intervals, but with greater relative fatality. For just in proportion to the extent and correctness of our knowledge concerning the efficient cause or causes of any particular disease will be our ability to modify or entirely counteract the effects. All the important questions relating to the establishment of Quarantines, and other sanitary regulations for the prevention of disease, in cities and isolated communities, can only be answered by a careful and rigid investigation of all those circumstances capable of affecting the health either of separate classes or of the whole community. Investigations by individual members of the profession and by special sanitary commissions, both in this country and Europe, have been made sufficient to show that as a general rule, diseases, whether endemic or epidemic prevail most and are most fatal in those parts of cities where the inhabitants are subjected to the combined influences of filth, dampness, and poverty. Hence great emphasis has been placed on the necessity of removing these circumstances, and the idea has been inculcated that such removal would be sufficient of itself to prevent the occurrence of severe epidemic or endemic diseases.

That the circumstances just alluded to are sufficient often to determine the development of typhoid and typhus fevers, dysentery, erysipelas, &c., in individuals exposed to them, I have no doubt. That by their debilitating influence on those habitually exposed to them, they act as predisposing causes of epidemic diseases, is also evident. Consequently there is generally a greater annual average mortality in districts where these circumstances exist than in those otherwise the same, but where these are absent.

It requires, however, but a moderately close search into the detailed history of epidemics occurring at the same place in successive years, or in different places during the same year, to show that the circumstances and conditions generally included under the head of filth, dampness, and poverty, are not alone, or all combined, sufficient to explain the origin and prevalence of such diseases.

A still smaller amount of research will show that their origin and spread cannot be *generally* traced to any species of importation, communicability or contagion. I say *generally*, because it must be admitted that in some instances the facts are so plainly and directly in favor of communication by importation in ships from infected places, that the conclusion cannot be easily avoided. Yet where one such unequivocal case occurs, a score of others are on record in reference to which no such origin can possibly be traced. Yet millions of money have been expended by municipal governments in establishing and enforcing Quarantine regulations under the confident expectation of preventing severe pestilences by cutting off their introduction from abroad. In view of the present uncertainty and defective knowledge on this subject, it is a duty incumbent upon the Medical Profession to enter upon a still more careful and extended investigation of this whole matter. In conducting this inquiry we should not start with the effort to collect such facts only as tend to corroborate or disprove some special theory; but we should first consider carefully, the various modes by which morbid causes, of whatever nature, may be made to act simultaneously on whole communities or particular parts of communities. If we reflect a little upon this preliminary question, we shall perceive that any cause capable of acting upon whole communities, must exist in the soil upon which they live, or in the water of which they drink, or in the air they breathe. In other words a cause, to act on many persons at the same time, must gain access to them through some one of those elements that are common to all. By doing this we obtain a basis for investigation; and we may next proceed to the inquiry whether particular changes in any of these elements invariably precede or accompany the prevalence of any one disease, or class of diseases. In pursuing this inquiry we shall find no difficulty in tracing a close relationship between certain appreciable conditions of the atmosphere, such as temperature and moisture, and the prevalence of some diseases.

Thus we find diarrhœas and dysenteries prevailing, more or less, during certain warm months of the year, in all cities and densely populated districts; however diverse may be the geological formations on which they are located.

Again, we trace with equal readiness the connection, to a certain extent at least, between certain geological formations and the prevalence of periodical fevers. But while we may easily fix upon certain conditions of soil and atmosphere which hold a pretty uniform relation to endemic diseases, or such as prevail habitually in particular localities, or during certain seasons of the year, the true *epidemics* appear and disappear so irregularly, at intervals so remote, and on geological formations so diverse that it is not easy to ferret out the laws which govern them, or the changes in the elements which precede and accompany their prevalence. We have indulged in these general observations, not with the intenton of pursuing the investigations to which they relate in all their bearing, but rather for the purpose of indicating their importance and of showing their connection with the observations which follow. As a member of the Committee on Epidemics and the Sanitary condition of this city, I must restrict my report on the present occasion mostly to a detail of facts as they have come under my observation during the past summer. But I trust the society will excuse me if I so far digress from the legitimate business of the committee as to compare these facts with those gathered during the two preceding summers, with a view of determining how far valuable conclusions may be drawn, in reference to the connection between certain atmospheric conditions and the prevalence of such epidemics, as have come under our observation. At the meeting of this society in June a report was made, containing a brief account of the prevalence of disease in the city from the beginning of the present year to that date. From that report and other sources, we learn that the monthly mortality thus far during the present year, has been as follows, viz.:

| | | | | | |
|---|---|---|---|---|---|
| January, 1855, whole number of deaths, | | | | | 123 |
| February, | " | " | " | " | 103 |
| March, | " | " | " | " | 124 |
| April, | " | " | " | " | 105 |
| May, | " | " | " | " | 90 |
| June, | " | " | " | " | 87 |
| July, | " | " | " | " | 239 |
| August, | " | " | " | " | 448 |
| Sept., | " | " | " | " | 310 |

It will be seen by this table that the smallest mumber of deaths occurred during the month of June, being less than three per day in a population of 80,000. The weekly reports of deaths show nearly the same low ratio of mortality during the first two weeks of July. During the third week, however, a pretty rapid increase took place, and during the fourth and fifth weeks the mortality reached near the maximum for the summer. Thus during the

| | | | | | | |
|---|---|---|---|---|---|---|
| First week in July, whole number of deaths was | | | | | | 22 |
| Second | " | " | " | " | " | 34 |
| Third | " | " | " | " | " | 74 |
| Fourth | " | " | " | " | " | 90 |

The weekly mortality for the month of August was as follows, viz.:

| | | | | | |
|---|---|---|---|---|---|
| First week | from | the 3d | to | the 10th, | 95 |
| Second | " | 10th | " | 17th, | 116 |
| Third | " | 17th | " | 24th, | 87 |
| Fourth | " | 24th | " | 31st, | 109 |

During the month of September, the mortality again decreased from week to week as follows:

| | | | | | |
|---|---|---|---|---|---|
| From September | 1st | to | 8th, | deaths, | 104 |
| " | " 8th | " | 15th, | " | 91 |
| " | " 15th | " | 22d, | " | 65 |
| " | " 22d | " | 29th, | " | 50 |

From these weekly details, it will be seen that the number of deaths rapidly increased from the middle of July to the 17th of August; then notably declined for one week, followed by an increase from the 24th of the latter month to the 15th of September; since which time the number has again steadily decreased. On examining the Meteorological records kept during the last three months, and comparing them with the foregoing tables of weekly mortality, some interesting coincidences may be noticed. The month of June was unusually cool, and the average degree of moisture in the atmosphere not above the ordinary standard; although to this there were some minor exceptions which will be noticed when we consider the prevalence of particular diseases. The first six days of July were all marked as clear, cool, and dry, except a slight sprinkling of rain on the morning of the third. During all these days the atmosphere contained less than the ordinary degree of moisture, and the wind was from the West, North-west, and North. The seventh was still clear and dry, but much warmer. The eighth was very warm, with south wind and copious showers of rain. The ninth was clear, extremely warm, and atmosphere *saturated* with moisture, with a continuance of wind from the South. The eleventh and twelfth were clear, cooler, and wind from the East. From the thirteenth to the nineteenth it was clear, except showers on the fifteenth and seventeenth. The atmospheric temperature and moisture were both high during the whole time, with the prevailing winds from the South and South-west. The morning of the nineteenth, was clear, warm and oppressive, with wind from the South and a moist atmosphere. It continued so until half past three o'clock, P. M., at which time the mercury in the Thermometer stood at 96° Fahrenheit. At that hour the wind suddenly changed and blew from the North-west and North, with so rapid a reduction of the atmospheric temperature, that at four o'clock, P. M., the mercury had sunk to 68° F.; thereby showing the downward range of 28° in half an hour. At six o'clock the same evening the temperature was only 62°. thus showing the extreme range of 34° in the short space of two and a half hours. It remained cool only about 48 hours, when the temperature again increased with the full medium degree of atmospheric moisture. On the 25th it was warm and rainy with the wind from the West. The 26th and 27th were recorded as "clear, but damp and oppressive," with the wind from the South-west. The remaining four days of the month were less hot, but the moisture of the atmosphere was but little if any less than during the preceding two days. The meteorological characteristics of the month of August are given as follows in one of the daily papers of the city, communicated by an intelligent druggist of this city, viz.:

"The promise of early summer has not disappointed us; and August has proved a cool, refreshing, and comfortable month. The mean temperature for the month has been 72°, more than 4° less than the average of August, 1854. The average height of the mercury at 7 A. M. was 66°; at noon 77°, and at 6 P. M. 72°.

"The mercury rose to 80° and over on thirteen days of the month, and fell below 60° on two days, the 17th and 18th. The hottest day was the 31st, when the mercury rose to 89° and the coldest on the 17th, when it descended to 52°. Thus the total range for the month was 37°. The average daily range was 12°, the highest on the 31st, 24°, the smallest on the 22d, when the mercury indicated the same degree (64°) throughout the day.

Refreshing rains have fallen on seven days of the month, twice accompanied with thunder, viz.: on the night of the 3d and the 15th. The sky has been clear on fifteen whole and seven half days, and cloudy on seven whole and nine half days.

The wind has blown from the North, on two whole and six half days, from the N. E. on five whole and five half days, and from the N. W. on one whole and two half days. It has blown from the South on one whole day and two half days, and from the S. E. on one whole day and five half days, and from the S. W. on five whole and six half days. It has blown from the East on three half days, and same from the West. The prevailing wind from the N. and Easterly quarters explain the coolness of the month, affecting favorably the health of the city, and the chief cause of sickness has been the sudden atmospheric changes, which have occurred on eight days of the month, more especially on the 20th, 21st, 24th, 25th, and 31st.

The summer of 1855 is ended; and all will gratefully contrast it with its predecessor the summer of 1854. JOSEPH WILLARD."

This account though sufficiently correct as far as it goes, is deficient in two particulars important to the Medical inquirier. It gives us averages of temperature, winds and rain, without stating to what extent any of these elements coincided at any one time; and it omits all information on the subject of atmospheric moisture.

If we should say that the seven rainy days were pretty equally distributed through the month; that each was preceded by two or three days of warm, clear, weather, and immediately followed by a North or North-east wind and two cool days, we should convey a pretty correct idea of the weather for the month. The coolest part of the month as indicated by the summary of Mr. Willard, as well as our records, was from the 17th to the 24th. With the exception of a very few days, the atmospheric moisture was above the average throughout the month. Its maximum degree was from the sixth to the fourteenth of the month. The meteorological characteristics of the first half of September corresponded very closely with those of August. On the seventeenth, at noon the temperature was 86° F.; but during the following night a violent storm of rain and wind from the North-east occurred, with a reduction of temperature to 62° F., on the 18th, and to 58° F., on the 19th at noon. From that time to the end of the month, the atmosphere was cool, moderately damp, and the prevailing winds from the North and East.

If we compare these meteorological details with the weekly mortality as already given, we shall find that every marked increase in the ratio of mortality was immediately preceded or accompanied by an elevated temperature, high degree of atmospheric moisture, and South or South-westerly winds.

For striking examples of this, note the rapid increase of mortality during the third week in July and the last week in August. On the other hand the continuance, for three or four days, of a low degree of temperature and moisture, with winds from the North and East, was uniformly followed by a decrease of mortality. This was strikingly illustrated during the week following the 17th of August, and again after the severe storm of the 17th and 18th of September. If we now turn our attention to the prevalence of particular diseases, we shall observe coincidences equally interesting with the foregoing. The month of June, as seen by reference to the table presents a lower ratio of mortality than any other month of the present year; yet it was during the latter part of that month, that those diseases commenced their prevalence which proved most fatal during the remainder of the summer. For several days preceding the 16th of June the atmosphere had been clear, dry, and pleasant. On the 16th it become more warm and damp, followed in the evening by copious showers with thunder, and a cool North-east wind.

During most of the 17th the atmosphere was filled with a dense cold fog, followed by rain again at night. The 18th, 19th and 20th, were clear and cool, but the atmosphere damp. On these days several fatal cases of cholera were reported at Bloomington, in the central part of the State, about one hundred miles South of this city. The morning of the 21st was quite warm and damp. At noon a shower of rain with wind from the West. The afternoon was clear, warm and damp. The 22d and 23d were cool and damp, with a dense chilling fog in the morning. From the 24th to the end of the month the atmosphere was moderately warm and moist with the prevailing wind from the South and South-west. Cases of acute dysentery began to occur, and rapidly increased in number from the 21st to the 23d; characterized by violent pains in the abdomen, tenesmus, and frequent discharges of mucus mixed with blood. During the same time attacks of diarrhœa and vomiting were frequent though rarely fatal. Some of these strongly resembled mild cases of genuine Cholera. From the 14th of June to the 8th of July the attacks both of dysentery and diarrhœa were less frequent, and generally easily controlled by medicine. On the 9th there was a remarkably rapid increase of both these diseases. Between the morning of the 9th and mid-day of the 10th, I saw in my own private practice twelve cases of dysentary, six of cholera morbus, all dating their commencement between the evening of the 8th, and the morning of the 10th. From the morning of the 11th to that of the 14th, I saw only two new cases of dysentery, and one of diarrhœa. From the 14th to the 19th, attacks of both diseases became more frequent. From this last date to the 15th of September, dysentery became *the prevailing* disease of the season, causing not less than thirty deaths in July, fifty-five in August, and forty in September. At no time during the past summer did the Cholera assume an epidemic form, and yet we had no period of three successive days, combining a warm and damp atmosphere, with South or South-west winds, without some fatal cases of this disease.

The whole number of deaths from Cholera, as near as could be ascertained, were in July 25, August 67, and September 44; making a total of 136. Attacks of ordinary intermittent and remittent fevers have been more frequent during the month of September than for several years past. The mortality from all the varieties of general fever, is reported for the month of August 10, and September, 21.

It is now three years since I commenced a careful series of observations on the atmospheric changes and the coincident prevalence of disease. These three years embrace the summers of 1853, '54, and '55 : and it may not be uninteresting to take a retrospective view of the prominent characteristics of each. With the exception of a few days about the middle of June, and a like period near the middle of August, the temperature of the summer of 1853, was about the average as deduced from a series of years. Both the exceptions named, were short periods of extraordinarily high heat. During all that summer, however, the atmosphere at this place and throughout the North-West, was remarkably dry. Nearly all the rains that occurred, were immediately followed also by a clear, cool, and dry atmosphere. The summer of 1854, presented features directly opposite. From the last of June to the first of October, the temperature was high and the atmospheric moisture much above the average standard.

The showers of rain that fell from time to time, were also followed immediately by a high degree of both temperature and moisture, creating an atmosphere at once oppressive and enervating. The prevailing winds were from the South and South-west. The summer of 1855, as you have already gathered from the details just given, has been charcterized by a low average temperature coupled with a high degree of atmospheric moisture; and with very few exceptions, the showers have been immediately followed by North and East winds, and sudden reduction of temperature.

The month of August, as already stated, gave an average temperature full 4° below that of the same month, in 1854. It will be seen that the three summers present striking meteorological differences. The differences in the prevalence of disease and the resulting mortality are equally striking.

The summer of 1853, for instance, was remarkably healthy throughout the North-West. The inhabitants of this city enjoyed an unusual exemption from the prevalence of epidemics and all other severe diseases. The ratio of mortality for the whole year of 1853, was only one for every fifty of the city population. On the other hand, the summer of 1854 was accompanied by one of the severest cholera epidemics that ever scourged our city; swelling the mortality for the entire year to the startling ratio of one in every nineteen of the population.

The summer of 1855, though free from the prevalence of any epidemic, and favoring our city with a ratio of mortality not above the average of a long series of years, was yet attended by decidedly more sickness, especially from dysentery and fevers, than that of 1853. If we estimate the mortality of the remaining three months of the year on the supposition that good health is continued, we shall have a total mortality for the year 1855 of two thousand one hundred; which is about one to every forty of the present population.

Thus we have in the first of the three years a ratio of mortality extremely low; in the second, extremely high; and in the third, about the average of ordinary healthy seasons.

If we enquire for the causes which have produced this diversity of results, we shall be compelled to look principally to the meteorological phenomena already detailed, for in every other particular the condition of the city has remained the same. There have been no important changes during these years, either in the cleanliness of the streets, the extent of sewerage, or in the habits and customs of the people. The only appreciable change in the hygienic or sanitary condition of the city, consisted in a more extensive supply of Lake water through the new Hydraulic works, which were put into operation early in the year 1854. The influx of new comers and the increase of population has been extremely rapid during all the years under consideration. Neither can it be claimed that the cholera epidemic, and consequent high ratio of mortality in 1854, was owing to any accidental importation of that disease from other locations. For there was the same tide of unrestrained immigration during the summer of 1853 as in 1854; and though some show of Quarantine regulations was adopted by the city authorities early in the summer of 1855, yet fatal cases of cholera were actually brought into the city at several different times.

One of the cases was mentioned in a report to the Society at its meeting in June. (See *N. W. Medical and Surgical Journal* for July 1855). Another occurred in the person of a Baggage Master on the Chicago and Mississippi R. R., who died in collapse at the Sherman House in this city, on the night of the 24th of June. Other similar cases occurred sufficient to show that there was no lack of importation from abroad; and certainly the cases occurring in our midst during the summer were sufficiently numerous to have propagated it, (if it were capable of propagation), as extensively as during any former season.

There was, therefore, nothing in the cleanliness of streets, the sewerage, the ventilation of houses, the supply of food or drink, or in the free communication with other locations which would cause the prevalence of disease during one of these seasons to differ materially from the others. From all my observations and reading during the past three years, I have arrived at the following conclusions in relation to the causes of cholera, diarrhœa, and dysentery:

First, that the origin and prevalence of these diseases depend directly on certain appreciable conditions of the atmosphere.

These conditions result from certain combinations of heat, moisture, and electricity, which are capable of affecting both the elementary properties of all the tissues, and the condition of the mucous and cutaneous surfaces of the body. The conditions most favorable to the development of cholera and serous diarrhœas are high temperature combined with a high degree of atmospheric moisture, and (in this locality) a South or South-west wind. At no period of the summers of 1854 and '55, did these conditions co-exist for three consecutive days without producing a marked increase of these affections. The same atmospheric conditions, coupled with frequent alterations of temperature from heat to cold, are most favorable to the production of dysentery; particularly of the inflammatory type. These two conditions were strikingly illustrated by the months of July and August, in the years 1854 and '55. The atmosphere during those months of the first named year was almost continuously hot, damp, and oppressive with the prevailing winds from the South. During those of the last named year, it was much of the time damp, but at no time hot more than three or four consecutive days without a sudden change to cold with North or North-east winds.

Second, that while certain atmospheric conditions constitute the efficient determining cause of the diseases under consideration, the local circumstances of filthy streets, imperfect drainage, confined air, over-crowding, the use of intoxicating drinks, &c., constitute important predisposing agencies, which though seldom sufficient alone to develope epidemic affections, yet always serve to render them, when once begun, more prevalent and fatal.

The foregoing details may appear to some tedious and unnecessary, but I only regret that my time will not permit me to make them still more minute and full. For one of the principal reasons why the facts of meteorology have heretofore thrown so little light on the causation of disease, is because observers have been too content with the obtaining of *mean* or *average* results for given periods of time, without patiently observing the daily variations and coincidences. It is easy to perceive that the same month in two successive years may give very nearly the same *mean*, both of temperature and atmospheric moisture, and yet these elements may be combined variously in different parts of that time.

